# The urgent need to empower rare disease organizations in China: an interview-based study

**DOI:** 10.1186/s13023-020-01568-5

**Published:** 2020-10-12

**Authors:** Xuefeng Li, Zijuan Lu, Jianyong Zhang, Xiangyu Zhang, Shu Zhang, Jincheng Zhou, Bingzhe Li, Li Ou

**Affiliations:** 1 Shenzhen Luohu People’s Hospital, The Third Affiliated Hospital of Shenzhen University, Shenzhen, 518001 People’s Republic of China; 2 Key Laboratory of Regenerative Biology, Guangdong Provincial Key Laboratory of Stem Cell and Regenerative Medicine, South China Institute for Stem Cell Biology and Regenerative Medicine, Guangzhou Institutes of Biomedicine and Health, Chinese Academy of Sciences, Guangzhou, 510530 People’s Republic of China; 3 The Sixth Affiliated Hospital of Guangzhou Medical University, Qingyuan People’s Hospital; State Key Laboratory of Respiratory Disease, Sino-French Hoffmann Institute, School of Basic Medical Sciences, Guangzhou Medical University, Guangzhou, 511436 People’s Republic of China; 4 School of Humanities, Tongji University, Shanghai, 200092 People’s Republic of China; 5 Jinhaishiji, 333 Jichanglu, Panzhihua, Sichuan 617000 People’s Republic of China; 6 School of Statistics, University of Minnesota, Minneapolis, MN 55455 USA; 7 Department of Oral Implantology, The Affiliated Stomatology Hospital of Kunming Medical University, Kunming, 650106 People’s Republic of China; 8 Center for Design and Analysis, Amgen Inc., Thousand Oaks, CA 91320 USA; 9 School of Electrical and Computer Engineering, Oklahoma State University, Stillwater, OK 74078 USA; 10 Gene Therapy Center, Department of Pediatrics, University of Minnesota, 5-174 MCB, 420 Washington Ave SE, Minneapolis, MN 55455 USA

**Keywords:** Rare disease, Patient organizations, Questionnaire, China

## Abstract

**Background:**

Each rare disease only affects a small number of population. However, a total of 7000 rare diseases may affect 10% of the population. Due to the severity and lack of rare disease awareness, rare disease represents a huge challenge for the healthcare system. In Western countries, patient organizations have been playing an integral role in raising awareness, advocating legislation, and supporting drug development. This study aims to assess the unmet needs of rare disease patient organizations in China, and identify their unmet needs, providing essential information for the government and legislators.

**Results:**

A total of 28 individuals representing 28 patient organizations in China were interviewed. Most organizations do not have official registration, employees, written standard operation protocol, or reliable financial resources. Misdiagnosis or delayed diagnosis is common, and treatment is often lacking. Due to the lack of financial resources, no organizations have been able to sponsor academic research, unlike their counterparts in Western countries. As to challenges, 71.4% of interviewees listed lack of rare disease awareness among the general public, while 67.9% selected lack of financial resources. Further, only 7.3% of these organizations received support from the government, and 28.6% received support from the general public. As to recommendations to the government, 82.1% of interviewees selected special insurance programs for rare diseases because rare diseases have been generally excluded from the national medical insurance programs. In addition, 78.6% of interviewees recommended to stimulate rare disease research, 75% recommended to import orphan drugs, and 71.4% recommended legislation of an orphan drug act, highlighting the urgent need of therapies.

**Conclusions:**

Due to lack of support and rare disease awareness, patient organizations in China are still in the early phase. To empower these patient organizations, the interviewees’ recommendations, including legislating orphan drug act and releasing official definition of rare diseases, should be considered by the government and legislators.

## Background

Rare diseases are diseases that affect a small number of population. Most rare diseases have identified genetic origins, and 50% of patients with rare diseases die before the age of 5 [1]. It was estimated that a total of 7000 rare diseases affect 300 million people worldwide. The definition of rare diseases differs as geographical regions of countries vary. In the United States, a rare disease is defined as any disease with a prevalence of fewer than 200,000 patients. The equivalent number in Japan is 50,000 patients, while in the European Union, the definition is any disease that affects 1 in 2000 people. Although rare diseases vary in etiology and clinical manifestations, most are associated with significant disease burden impacting physical and mental abilities, as well as life expectancy [2–4]. As a huge group, rare diseases constitute a significant challenge for the healthcare system and the economy, and thus should not be neglected by the society and the government [5, 6].

Patient organizations have been playing a critical role in forming supportive groups, advocating to reduce health disparity and discrimination, as well as building a sense of community [7]. Moreover, patient organizations are the driving force of the rare disease ecosystem and have a role at all levels, including supporting patients, initiating and sponsoring research, prompting legislation, generating educational materials, and raising public awareness [8]. It is essential to evaluate the status quo of patient organizations in China, a key player in the ecosystem, and identify their unmet needs and ways to empower them. Therefore, an interview-based study was conducted, and recommendations were made for consideration of policymakers.

## Methods

### Ethics, consent, and permission

The study was approved by the Institutional Ethics Committee of Guangzhou Medical University. Potential interviewees were invited to participate this study, and only those who signed the informed consent participated in this study. All the interviewees acknowledged: (1) the affiliation of the investigators; (2) the sponsor of the study; (3) the objectives of the study, (4) that the information collected will only be used for academic research; (5) that they will participate in this study anonymously; (6) that they can decline to answer any of the questions; (7) that they can quit the study at any time; (8) that the results will be published in a scientific journal without seeking their approval of the manuscript; and (9) that they will not be paid for participating in this study.

### Data collection and analysis

The interviewees, usually a key person in a patient organization, were recruited through online advertisements or personal references. An online webinar was held to brief potential participates on the background of the study and answer their questions. Then, the investigators contacted potential participants to confirm the interests and schedule the interview. Finally, the interviews were performed via a 1 to 2-h phone talk. The interviewees are independent in providing answers to a list of 18 questions, most of which are open-ended questions (Additional file 1). All interviews were conducted by the investigators between March to May 2020. The interviews were audio-recorded with the interviewees’ permission and transcribed verbatim for further analysis. Common themes from these transcripts were analyzed in this study.

## Results

### Information about patient organizations

#### How interviewees got involved with a rare disease

A total of 28 interviewees, representing 28 patient organizations, participated in this study. Information about the 28 interviewees and their organizations is summarized in Table 1. Most (25/28, 89.3%) interviewees got involved because they or their family members had a rare disease. Twelve out of 26 respondents (46.1%) were funders of these patient organizations. Two organizations were started by individuals who were not personally affected by rare diseases.

**Table 1:** Information of patient organizations

Organizations	Disease of interest	On the List of Rare Diseases?	Relationship to a rare disease?	Is the interviewee the founder?	Current members	Total patients in China	Reliable financial resource	Official registration?	Employee?	Office space?	SOP?
Anning's Mother PKU Chat Group	Phenylketonuria	Yes	Family	Yes	1000	140,000	No	No	No	No	No
MPS I Chinese Patients Community	Mucopolysaccharidosis type I	Yes	Self	Yes	Not sure	Not sure	No	No	No	No	No
Cushing Syndrome Community	Cushing syndrome	No	Self	Yes	800	4000	No	No	No	No	Yes
Nanjing Rare Disease Help Center	Acromegaly	No	No	Yes	800	100,000	No	Yes	No	No	No
Usher Syndrome Chat Group	Usher syndrome	No	Self	No	Not sure	30,000–40,000	No	No	No	No	No
Beijing Zhi'ai DMD Help Center	Duchenne muscular dystrophy	Yes	Family	No	5000	70,000	No	Yes	Yes	No	No
Sichuan Huntington's Disease Community	Huntington's disease	Yes	Self	No	600	4000–5000	No	No	No	No	Yes
Gaucher Disease Patient Club	Gaucher disease	Yes	Self	No	300	400	No	No	No	No	No
Body Odor Chat Group	Unidentified diseases that cause body odor	No	Self	Yes	2000	20,000–50,000	No	No	No	No	No
Acromegaly Communication Center	Acromegaly	No	Self	Yes	600	100,000	NO	No	Yes	No	Yes
Shanghai ALD Mutual Help Group	Adrenoleukodystrophy	Yes	Family	No	1000	Not sure	No	No	No	No	No
CAH & AHC Help Center	Congenital adrenal hyperplasia, adrenal hypoplasia congenita	Yes	Family	Yes	10,000	100,000	No	No	No	No	No
SPE Patients Chat Group	Symmetrical progressive erythrokeratoderma	No	Family	No	Not sure	1000–10,000	No	No	No	No	No
VWD Patient Community	Von Willebrand disease	No	Self	Yes	200	160,000	No	No	No	No	No
CGL Patient Community	Chronic Granulocytic Leukemia	No	Self	Yes	Not sure	Not sure	No	No	No	No	No
LCA Patients Club	Leber congenital amaurosis	No	Self	No	300	30,000	No	No	No	No	Yes
Shandong Osteogenesis Imperfecta Chat Group	Osteogenesis imperfecta	Yes	Self	No	600	Not sure	No	No	No	No	No
Pompe Patients Help Center	Pompe disease	No	Self	No	400	5000	No	No	Yes	No	Yes
LNS-China	Lesch–Nyhan syndrome	No	Family	Yes	15	200	No	No	Yes	No	No
Chinese Fabry Patients Club	Fabry disease	Yes	Self	No	300	1000	No	No	No	No	No
Zhuo Wei Chang Dao	Dravet syndrome	Yes	Family	Yes	1000	20,000	No	No	No	No	Yes
Beijing Zhengyu MPS Disease Center	Mucopolysaccharidoses	Yes	Family	No	400	2000–3000	No	No	No	Yes	Yes
MMA Patients Community	Methylmalonic acidemia	Yes	Self	No	800	50,000	No	No	No	No	No
Butterfly Baby Help Center	Epidermolysis bullosa	Yes	Self	No	300	2000	No	Yes	No	No	No
Chongqing Hemophilia Patients Club	Hemophilia	Yes	Self	No	3000	100,000	No	No	No	No	Yes
Seven-Pansy Rare Disease Community	All rare diseases	N/A	No	Yes	10,000	20,000,000	No	Yes	Yes	No	Yes
Henan Neurofibromatosis Patients Club	Neurofibromatosis	No	Self	No	500	100,000	No	No	No	No	Yes
TSC Patient Communication & Help Center	Tuberous sclerosis complex	Yes	Self	No	3000	100,000	No	No	No	No	No

SOP, standard operation protocol

#### Organizations

All organizations were started between 2012 to 2019. Only one, Seven-Pansy Rare Disease Community, which is a national umbrella organization for all rare disease patients in China, has a website. Most interviewees believed that financial and technical difficulty was the major reason that their organizations did not have websites. All 28 organizations were mainly formed through online chat group (initially QQ, now WeChat). Nowadays, WeChat has over 1.1 billion active users, and constituted an essential part of social life in China. Some interviewees expressed the concern over the usage of WeChat. As an instant communication tool, it is difficult to categorize, store, and search for useful information. Seven patient organizations had WeChat blogs that publish useful materials, which can be read and commented on. WeChat blogs are easier to manage than websites, but still create some difficulty for patient organizations. Only 5 organizations were officially registered. One interviewee explained that official registration would not provide extra benefits. Only 5 organizations had full-time or part-time employees, only 1 organization had an office, and only 9 organizations had written standard operation protocols. Most interviewees attributed this to the lack of funding sources. Proper training and financial support are needed. None of these organizations had reliable funding sources, while most (17/28, 60.7%) did not have any funding sources. Out of the 10 organizations (35.7%) that had some funding sources, five (17.9%) relied on the founders’ personal incomes, three (10.7%) received public donations through crowdfunding, and two received donations from corporations. Some interviewees believed that this was because corporations were not motivated enough to donate, and rare disease awareness was lacking among the general public. One interviewee commented: ‘Unlike China, many patient organizations in the USA received donations from corporations, pharmaceutical or other industries, because these corporations would receive tax benefits.’ Another interviewee mentioned that ‘Some corporations in the USA have a special team to donate when approaching the tax filing date so that they can get a tax cut.’

### Rare diseases associated with patient organizations

#### Diagnosis

Genetic testing is largely unavailable or unaffordable, and there lacks genetic counseling. According to these interviewees, patients and families often receive a report full of jargon and technical terms without sufficient explanations or firm conclusions. It usually turns out that ‘the genetic test report generated more questions than it answered.’ All interviewees expressed concern over misdiagnosis and delayed diagnosis. Newborn screening was believed to be essential, and some interviewees initiated campaigns to implement newborn screening programs in China. Through one interviewee’s efforts, MPS I was included in a newborn screening panel in several hospitals in Beijing. She commented that ‘Although national newborn screening is not realistic now, this is an initial step.’

#### Prevalence

Although some academic researchers performed epidemiology studies for some rare diseases [9, 10], few national prevalence studies have been conducted [11]. Interviewees estimated the number of patients based on their judgment (Table 1). In 2018, the Chinese government issued the First National List of Rare Diseases, including 121 rare diseases [12]. However, there are no official definitions of rare disease in China. Therefore, the total number of rare disease patients remains unknown. It was estimated that there were 16.8 million rare disease patients in China. However, it may be a significant underestimation. In the United States, there are 30 million rare disease patients, constituting 10% of its population [13]. It was estimated that the total number of rare disease patients in the world to be 300 million, constituting approximately 4% of the world population [14]. An estimated 5000 to 8000 rare diseases have been identified worldwide, affecting approximately 6 to 8% of the population [15]. Considering the 1.4 billion population of China, the total number of rare disease patients may be significantly over 16.8 million. A rare disease usually causes significant economic and psychological burden to a family [4], so the number of people impacted by rare diseases would be even more. Additionally, since the government has released a list of 121 rare diseases, the upper limit of what constitutes a rare disease in China could be derived from the prevalence of the diseases on this list.

#### Treatment and management

A previous study showed that most rare disease patients had experienced difficulty in access to treatment, and fewer than 10% have received disease-specific treatment [11]. None of the rare diseases associated with these organizations are curable, only 10 (37.0%) have disease-specific treatment. Even when there is an option, the delayed diagnosis or misdiagnosis would have already costed patients the opportunities to receive timely treatment. For instance, MPS I disease can be treated by stem cell transplantation, which can halt neurological deterioration and improve quality of life if performed before age 2. One organization, Seven-Pansy had been trying to import orphan drugs through a government-sponsored special project called Hainan Boao Lecheng International Medical Tourism Pilot Zone [16]. This project aims to import drugs due to urgent clinical needs for us in designated medical institutions. Based on patients’ needs, Seven-Pansy had drafted a list of orphan drugs for the Boao project.

### Activities of patient organizations

#### Outreach to patients

For most patient organizations, reaching out to patients or their families were initially difficult. Most patient organizations were started by several families that acquainted with each other in hospitals. Then, the core families took the initiative to contact others. Currently, the major source is the internet. Another source is referrals from other patients in hospitals. For instance, at Xinhua Hospital, Shanghai, where many patients with mucopolysaccharidoses, received transplant, patient families lived there for a while for recovering from transplant surgery. They formed an online chat group, which accumulated 500 members over time. Five interviewees mentioned referrals from doctors as a source of recruitment. Only 46.4% (13/28) of interviewees had been asking the family history of new members to identify carriers and potential patients. Since genetic counseling is not widely available in China, and people generally lack the genetics knowledge, it is important to understand the family history to identify carriers and potential patients. Otherwise, the diagnosis of patients in those families may be delayed, and timely treatment cannot be conducted. In some diseases, for instance, Fabry disease, an X-linked recessive disease, female carriers would have some symptoms that need to be treated as well [17].

#### Rare disease awareness

**Fig. 1:**
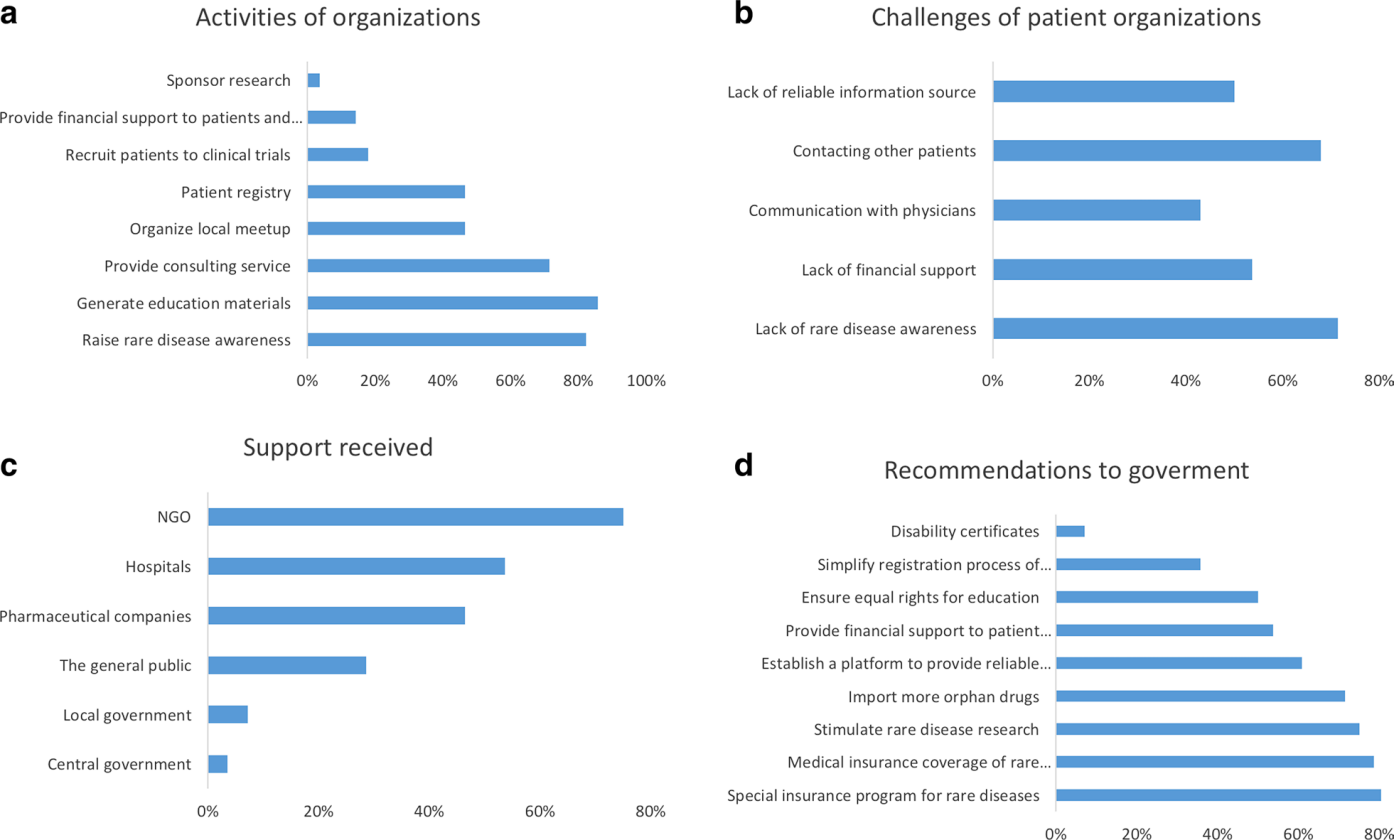
Activities, challenges, support received, and recommendations of patient organizations. Activities (**a**), challenges (**b**), support received (**c**), and recommendations (**d**) were shown, respectively. Information was collected through one-on-one interview with leaders from 28 patient organizations in China

Large organizations in Western countries, for instance, the National Organization for Rare Disorders (NORD) in the United States and the European Organization for Rare Diseases (EURORDIS) in Europe, have been contributing significantly to raising rare disease awareness. The Rare Disease Day event, initiated by EURORDIS, has now become a well-known event worldwide. However, such a national platform or information hub with a similar influence still lacks in China. As shown in Fig. 1a, 23 of 28 organizations (82.1%) had activities to raise rare disease awareness, mainly through the internet. Several interviewees mentioned that they had organized members to hand out pamphlets on the street. There has been increasing rare disease awareness in China [9, 18], which may be attributed to the efforts of patient organizations.

#### Patient support

Since most of these organizations had no reliable funding source, only 4 organizations (14.3%) were able to provide financial support to patients and families. A total of 24 organizations (85.7%) provided education materials with input from physicians and researchers. However, none of these organizations were able to generate educational materials to guide physicians, which have been attempted by organizations in Western countries. For instance, NORD issued a comprehensive physician guide to rare diseases [8]. Most organizations (20/28, 71.4%) provided consulting services to patients and families. This was mainly through expert patients or occasionally through invited physicians. Another form of patient support is local meetup, which had been performed by 13 organizations (46.4%). Three interviewees commented that local meetups could provide more direct communication, which is important to relieve stress and seek comfort from each other.

#### Research

In Western countries, many patient organizations sponsor academic research with the main focus on therapy development [19, 20]. For instance, many organizations, such as NORD (USA), Association Française contre les Myopathies (France), Children Living with Inherited Metabolic Disorders (UK), Sanfilippo Children’s Foundation (Australia), have annual grant programs to support rare disease research. Also, several patient organizations in Europe, Australia, and the United States contributed millions of dollars and helped ABO-101 and ABO-102, two gene therapy products for MPS III diseases, reach the clinical trial stage. In contrast, patient organizations in China, mainly due to the lack of financial support, have been not able to sponsor academic research. Out of these 28 organizations, 5 (17.9%) had helped to recruit patients for clinical trials, and 13 (46.4%) maintained patient registries. Although it is essential to have a national registry for rare diseases, these organizations have not been able to establish one. In Western countries, there also has been a transition of patients as participants or financial supports into collaborators in research. Members of patient organizations have been actively involved in academic research and drug development, resulting in peer-reviewed publications and patents [21, 22]. This is rarely seen in China, and none of the interviewees in this study had played such a role in academic research.

### Unmet needs of patient organizations

#### Challenges

Most organizations (20/28, 71.4%) mentioned the lack of rare disease awareness among the general public as a major challenge. Also, 19 organizations (67.9%) mentioned the lack of financial sources as a major challenge. Fifteen organizations (53.6%) believed contacting other patients was challenging, 14 (50%) selected communication with doctors, while 14 (50%) mentioned the lack of reliable information source (Fig. 1b). Three interviewees mentioned that the availability and affordability of orphan drugs were poor.

#### Support received

Only 1 (3.6%) received support from the central government, and only 2 received support from the local governments (Fig. 1c). Only 8 (28.6%) received support from the general public. The major source of support these organizations received came from non-profit organizations (21/28, 75%), pharmaceutical companies (14/28, 50%), and hospitals (15/28, 53.6%). These results indicated a significant lack of support from the government and the general public.

#### Interviewees’ recommendations to the government

As shown in Fig. 1d, 23 interviewees (82.1%) selected ‘special insurance program for rare diseases or inclusion of rare diseases into the Critical Illness Insurance Program’. Twenty-two interviewees (78.6%) selected ‘stimulate rare disease research’, 21 (75%) selected ‘import more orphan drugs’, and 21 (75%) selected ‘establish a platform to provide reliable information’. Also, 20 interviewees (71.4%) selected ‘legislate orphan drug act’, 17 (60.7%) selected ‘provide financial support to patients’, and 15 (53.6%) selected ‘provide financial support to patient organizations’. Additionally, 14 (50%) selected ‘address discrimination in school’, 10 (35.7%) selected ‘simplify registration process of organizations’, and 2 (7.1%) selected ‘disability certificate’. One interviewee commented that ‘raise public awareness so that patients can be respected, understood, and equally treated by employers’. He further explained: ‘some local governments had written policies to exclude patients with acromegaly while hiring civil servants.’ This practice of the local governments is directly against the Law of the People's Republic of China on the Protection of Disabled Persons [23].

## Discussion

Patient organizations have been playing a critical role in advancing the field of rare diseases by advocating patient’s voices and addressing the unmet needs of patients. As shown in this study, patient organizations in China have been contributing to the filed by generating educational materials and providing support to patients. However, these organizations in China have not been playing an active role in advancing legislative agenda. In contrast, the NORD from the United States organized a series of headline events that raised wide public awareness and prompted the legislation of the world’s first orphan drug act [24, 25]. This act provides incentives, including market exclusivity, tax benefits, fast track approval, and government subsidy, to stimulate orphan drug development. These incentives have been to be particularly appealing to pharmaceutical companies [26, 27]. The number of orphan drugs increased from 38 in 1983 to over 500 in 2018. Therefore, similar acts have been established in many other countries and districts, including Japan, European Union, Singapore, South Korea, and Australia [28, 29]. Also, patient organizations in China were generally not active in academic research. In contrast, EURODIS and Canadian Organization for Rare Diseases (CORD) have been playing an integral role in spearheading rare disease and orphan drug legislation, patient support, raising awareness, and sponsoring academic research. In addition, most patient organizations in China do not have websites, enough manpower, official registration, and reliable funding source. Therefore, compared with patient organizations in the United States, Europe [30], Australia [31], and India [32], patient organizations in China are still in the early phase. This was believed to be due to the lack of support and rare disease awareness in China.

Clearly, there is an urgent need to empower patient organizations in China and thus advance this field. Key recommendations that outline top priorities that the government and legislators should consider are listed as follows. These recommendations are ordered based on results from Fig. 1d (a multiple choice question) and answers to an open-ended question about the most urgent issue that needs to be addressed.

1. *Legislate an Orphan Drug Act to stimulate orphan drug development*. Witnessing the benefits brought by the Orphan Drug Act of 1983, there is a high interest in legislating a similar orphan drug in China, which provides orphan drug exclusivity, tax incentives, market exclusivity to motivate orphan drug development.
2. *Issue official definition of rare disease*. As shown in Table 1, the First List of 121 Rare Diseases does not include all rare diseases studied here, not to mention the total of 7000 rare diseases. Many interviewees mentioned the need to include their rare diseases into a second list, or to issue an official definition of rare diseases like other countries.
3. *Raise rare disease awareness to shorten the diagnosis odyssey*. The lack of rare disease awareness was believed to be a major reason that patient organizations in China were underdeveloped and unsupported. Rare disease awareness among parents and physicians may significantly shorten the diagnostic odyssey.
4. *Implement prenatal and newborn screening of a subset of rare diseases*. Newborn screening is largely unavailable in China, resulting in delayed diagnosis and treatment. Despite efforts from some organizations, there is still a need to improve the availability and affordability of prenatal and newborn screening.
5. *Establish special insurance programs to cover the cost of the treatment and long-term management of rare diseases*. Most interviewees expressed concern over the high cost and low insurance coverage of orphan drugs, especially gene therapies.
6. *Issue regulations to protect rare disease patients from discrimination in education and employment*. Concern over the inequality in education and employment were also common among interviewees. Implementation of regulations to protect patients’ rights were expected to be able to significantly improve the quality of life and social status of patients.
7. *Import more orphan drugs*. Due to the lack of domestic R&D efforts in orphan drugs, it is a common situation that orphan drugs available to patients in other countries were not available in China. Although there has been attempts from the government to import more orphan drugs, the demand is still high.

## Conclusions

Rare disease patient organizations have been playing a critical role in advocating patients’ voice, prompting legislation, raising awareness, educating and supporting patients, and sponsoring academic research. However, unlike their counterparts in Western countries, patient organizations in China are less developed due to lack of support and rare disease awareness among the public. This study evaluated the current status of 28 patient organizations in China through an interview-based study and identified their unmet needs. These unmet needs include lack of financial support, reliable information, therapies, and rare disease awareness. Therefore, recommendations have been made for the policymakers to consider to empower patient organizations in China. Specifically, legislation of orphan drug act and rare disease definitions are highly recommended.

## Supplementary information

**Additional file 1:** List of interview questions.

## Data Availability

The interview transcripts are not publicly available due to the agreement between the investigators and the interviewees.

## Abbreviations

NORD: National Organization for Rare Disorders
EURORDIS: European Organization for Rare Diseases
CORD: Canadian Organization for Rare Diseases
