# Supplementary material for: The urgent need to empower rare disease organizations in China: an interview-based study
Source: Orphanet J Rare Dis. 2020 Oct 12;15:282. doi: 10.1186/s13023-020-01568-5 (PMC7552513; doi:10.1186/s13023-020-01568-5)
Supplement: Supplementary file 1 — Additional file 1: List of interview questions. [file 13023_2020_1568_MOESM1_ESM.docx]

**问题列表 (List of questions)**

1. 您所在组织全称是 (What is the full name of your organization?)

2. 您本人或者家人患有这种罕见病吗？(How do you get involved with this rare disease?)

3. 这种疾病可以治愈吗？有什么治疗手段？有什么控制手段（如特殊饮食和辅助措施）？(Is there a cure, therapy, treatment, or management option for this disease?)

4. 您是否是组织创始人？(Are you the founder?)

5. 您的组织是什么时候成立的？(When was it founded?)

6. 目前您的组织有多少病友？ (How many members now?)

7. 全国大概有多少患者？(How many patients in China?)

8. 您的组织如何招募新的患者？(How does your organization recruit patients?)

9. 您的组织是否会询问患者的家族病史以便找到携带者或者可能的患者？(Does your organization ask about family history to identify carriers or potential patients/)

10. 您的组织主要经费来源是？稳定吗？(Does your organization have reliable financial resource?)

11. 您的组织主要工作是什么？组织了哪些活动？(What kind of activities has your organization done?)

12. 您的组织是否有正式注册，全职员工，固定办公场所， 和成文的工作流程？(Does your organization have official registration, employees, work place, and written standard operation protocol?)

13. 您的组织遇到了哪些方面的困难？(What kind you challenges has your organization been facing?)

14. 您的组织从以下机构得到了帮助？中央政府, 地方政府, 公众, 药企, 医院,公益组织. (Has your organization receive support from the central government, local government, public, pharmaceutical companies, hospitals, or NGOs?

15. 您有哪些政策建议？

简化患者组织注册流程

给患者组织的经济补助

罕见病特别医保或者直接纳入大病医保

给患者的特殊补助和救济

残疾人证

加大力度引入国外罕见病药物

孤儿药法案（给药企提供更多激励研发罕见病药物）

普及新生儿筛选

学校照顾罕见病儿童利益

鼓励罕见病相关研究

建立罕见病信息平台促进信息流通

(Do you have any recommendations to the policy-makers? e.g., legislate Orphan Drug Act, special insurance program for rare diseases or inclusion of rare diseases into the Critical Illness Insurance Program, stimulate rare disease research, import more orphan drugs, establish a platform to provide reliable information, provide financial support to patients, provide financial support to patient organizations, address discrimination in school, simplify registration process of organizations, disability certificate.)

16. 您对《罕见病目录》有什么看法？与您相关的疾病被列入这一目录会带来什么具体的影响？(Any comments on the List of 121 Rare Diseases?)

17. 国内有哪些医院和专家在这方面比较有经验？(Do you know any doctors and experts in the rare disease that your organization is associated with?)

18. 您的组织有什么最紧迫的诉求吗？(What is the most urgent issue that needs to be addressed?)
